# 

**Published:** 1892-07

**Authors:** 


CONTENTS

Vol. 39. No. 7.

| | PAGE. |
|---|---|
| Talks with Dr. Mandeville... .. | 145 |
| Approved Criminality......... | 147 |
| Longevity of the Sexes. ....... | 149 |
| Spiritualism Pure and Simple.. | 150 |
| Death from Fright ............ | 151 |
| Stranger than Fiction .... ... | 152 |
| The Nurse—Lying-in Chamber | 154 |
| Another Famous Man saw Spirits | 156 |
| Hints regarding Children....... | 157 |
| Milk as a Diet... ............. | 158 |
| Bee-Keeping .................. | 159 |
| Care of the Ears .............. | 160 |
| Beer Drinking.................. | 161 |
| Destiny ...................... .. | 162 |
| A Married Man's First Duty.... | 163 |
| Pure Air and Life............. | 163 |
| Miscellaneous ................. | 164 |
| Literary.......... ........... | 167 |
| A Great Man.................. | 168 |

INDEX TO ADVERTISEMENTS OF HALF A PAGE AND OVER.

| | PAGE. |
|---|---|
| Christian Life.... .......... | I |
| Magic Fuel Co. ......... ..... | II |
| Bovinine ................... | IV |
| Ayer's Cherry Pectoral ...... | V |
| Hall's Journal Premiums. ... | VII |
| Washington Improvement Co. | VIII |
| Herba Vita..... .......... . | X |
| Royal Baking Powder....... | XI |
| Buffalo Lithia Water...... . | XI |

